# Ag-AgCl Nanoparticles Fixation on Electrospun PVA Fibres: Technological Concept and Progress

**DOI:** 10.1038/s41598-019-51642-7

**Published:** 2019-10-29

**Authors:** Zuzana Vilamová, Zuzana Konvičková, Petr Mikeš, Veronika Holišová, Pavel Mančík, Edmund Dobročka, Gabriela Kratošová, Jana Seidlerová

**Affiliations:** 1 Nanotechnology Centre, VŠB – Technical University of Ostrava, 17. listopadu 2172/15, Ostrava, 708 00 Czech Republic; 2 ENET Centre, VŠB – Technical University of Ostrava, 17. listopadu 2172/15, Ostrava, 708 00 Czech Republic; 3 Department of Chemistry, Faculty of Science, Humanities and Education, Technical University of Liberec, Studentská 5, Liberec, 461 17 Czech Republic; 4 IT4 Innovations, VŠB – Technical University of Ostrava, 17. listopadu 2172/15, Ostrava, 708 00 Czech Republic; 5 Institute of Electrical Engineering, Slovak Academy of Sciences, Dúbravská cesta 9, 841 04 Bratislava, Slovak Republic

**Keywords:** Nanocomposites, Characterization and analytical techniques, Nanoparticles

## Abstract

Polymer-metal based material with unique 3D structure is an attractive substrate for the development of biomedical applications. A novel preparation of the composite from polymer fibres and silver nanoparticles has been designed through: (1) preparation of silver nanoparticles by phytosynthesis and (2) incorporation of these nanoparticles in a fibrous membrane prepared by electrospinning. The nanoparticle biosynthesis was performed in a pure environmental-friendly, easy, static, bottom-up *in vitro* regime using *Tilia* sp. leachate. TEM and XRD depict the formation, stabilisation and encapsulation of crystalline silver (14 ± 9 nm) nanoparticles (NPs) in one simple step with low tendency to aggregate. We achieved successful incorporation in the uniform electrospun 221 ± 24 nm poly(vinylalcohol) fibres, and this confirms the possibility of its use in the biomedical field. Both SEM with EDX and TEM analysis determined fibre uniformity with the presence of silver NPs, and ICP-AES confirmed the relatively similar metal concentration throughout the triplicate measurement of fibre structures on the 2 × 2 cm area in the following manner: 0.303 ± 0.018 wt. %, 0.282 ± 0.017 wt. %, and 0.281 ± 0.017 wt. %. Our hypothesis is based on previously verified preparation of active silver NPs and the easily prepared PVA electrospun fibres which act as a water soluble matrix. The simple methodology of incorporating biosynthetically prepared NPs in the PVA fibers highlights the effectiveness of this material, with simple release from water-soluble PVA and final activation of the prepared NPs.

## Introduction

The attractiveness of nanoscale inorganic forms in material and biological fields has started an interest in technological research. Their main properties, such as composition, shape and 3D hierarchy as well as their incorporation to more complex materials could be used in different applications^1^.

Biosynthesis of metallic nanomaterials by bacteria^2^, cyanobacteria^3^, algae^4^, microscopic fungi^5^, live plants and/or waste biomass^6^ and different plant extract types has been confirmed, and the individual biomasses contain large numbers of organic compounds composed of positively and negatively charged functional groups. Examples of negatively charged groups include hydroxyl (-OH), amino (-NH_2_) and carboxyl (-COOH) groups; and metal ions (Ag^+^ or Au^+^^3^) can be reduced to zero-valent or different form when the biomass and a metal salt precursor are mixed.

Silver has an important role in two scientific areas: (1) as an antimicrobial agent in medicine^7^ and (2) heterogeneous catalysis in degrading organic pollutants^8^. While nanosilver’s promising antimicrobial properties are advantageous in the burgeoning increase in multi-drug-resistant bacteria from global antibiotic overuse, its precise effects are undetermined^9^. However, novel catalytic properties, including the greatly enhanced reactivity and selectivity, have been reported for nanosilver^8^ and NP integration in the catalytic process transforms initial reactants rapidly, thus significantly reducing reaction time.

Promising application potential is also clear in the area of macromolecular chemistry. Overlap in the production of polymeric materials and materials based on the polymer-metal platform enable wide-spread application in material engineering, medicine and biotechnology^10^. The electrospinning is an technique using a high voltage electrostatic field to charge the surface of liquid solutions. This is especially important for these polymers, where the technology is divided into ‘capillary’ and **‘**needleless’ categories, dependent on the extracted fibre’s location^11,12^. Although capillary electrospinning is limited by the amount of injected polymer, the needleless technique has high fiber production from the free surface of the polymer via self-organization^13^.

Scientists have recently focused on artificial dressings prepared by this electrospinning method. These are produced from natural polymeric materials, including collagen and gelatin and from synthetic polymeric materials such as poly(vinylalcohol) (PVA) and poly(lactic acid) (PLA). These artificial dressings should meet the following criteria: (1) be harmless for humans; (2) mechanically stable; (3) biodegradable and (4) provide an appropriate environment for tissue repair. They must also fulfil the essential requirements of enabling rapid hemostasis and have effective antibacterial properties^14^. The antibacterial electrospun nanofibres are usually produced by incorporating antibiotic materials such as ofloxacin^15^, Ag NPs^16,17^ or other metallic and metal oxide NPs^18^.

We previously discovered the benefits of biosynthetically prepared Ag-AgCl NPs^19^ and this study now elucidates novel methodology for preparation of composite materials. Herein, we focus on two main steps: (1) biosynthetic preparation of Ag-AgCl NPs using *Tilia* sp. extract and (2) preparation of composite material with PVA electrospun fibres incorporating Ag-AgCl NPs (PVA-Ag). The hypothesis was based on previously verified preparation of active Ag-AgCl NPs^19^ and easy-to-prepare PVA electrospun fibres. PVA is a semi-crystalline and water-soluble polymer with chemical and thermal stability, and it is also non-toxic, non-carcinogenic and biocompatible with excellent electrospinning ability^20^.

The novelty of our preparation is proven in the simple methodology of biosynthetic NPs integration into the PVA fibres. The major benefits of this material are simple NP release to the nearest surrounds due to PVA water-solubility and final activation of the prepared NPs.

## Results and Discussion

### Prepared Ag-AgCl nanoparticles structure and phase analysis

**Figure 1:**
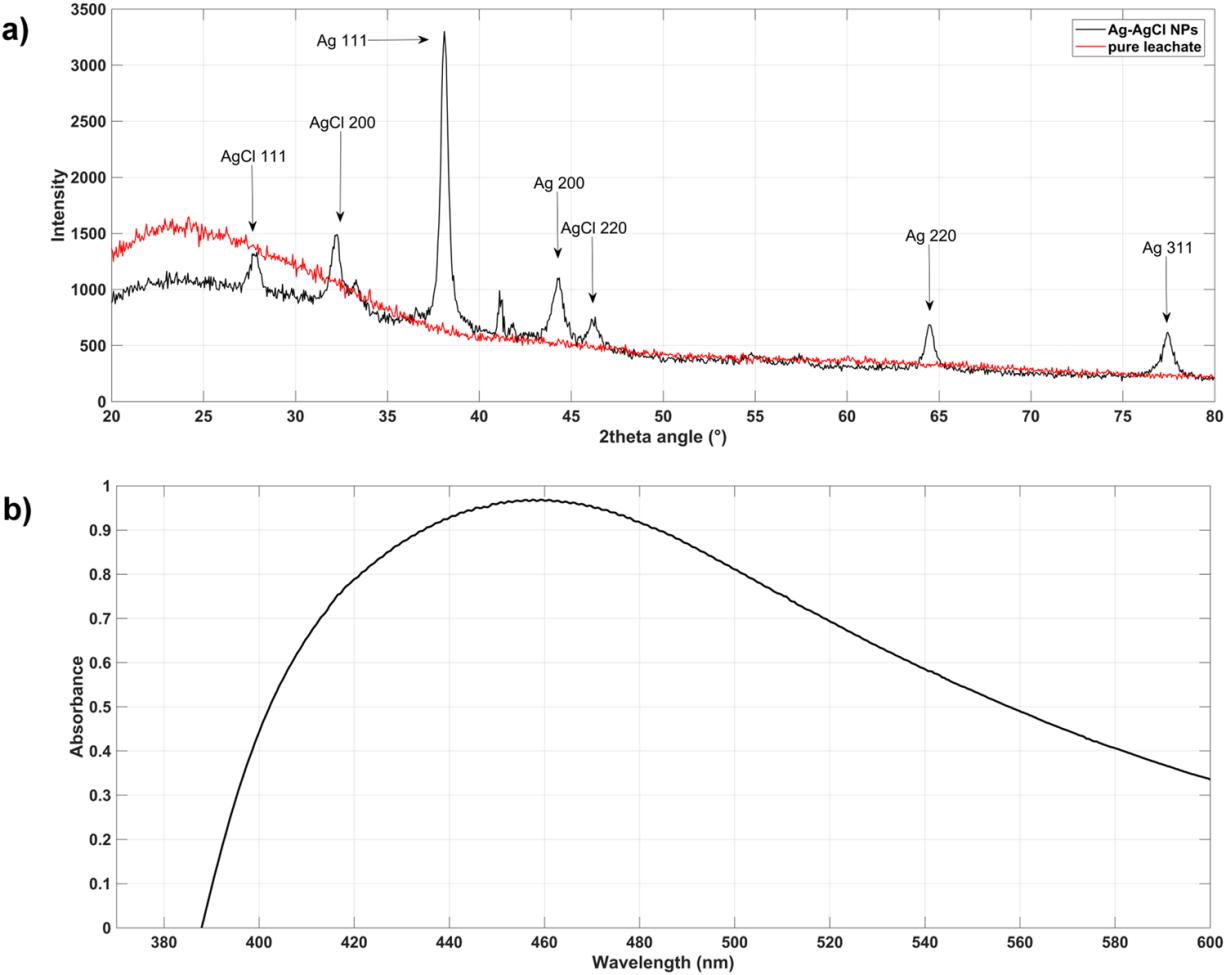
(**a**) UV/VIS spectroscopy confirmed characteristic absorption peaks at 460 nm for Ag-AgCl NPs; (**b**) diffraction pattern of Ag-AgCl NPs: the peaks and the strongest lines for silver chloride and cubic silver are clear.

XRD analysis confirmed the crystalline character of Ag-AgCl NPs in the colloid sample and the XRD pattern was recorded in the 2*θ* 20–80° range (Fig. 1a). The pure silver (PDF 00-004-0783) and chlorargyrite AgCl (PDF 01-085-1355) crystalline phases were identified, and both are cubic with *fcc* lattice and Fm3m space-grouping. Figure 1a also highlights the indices of the strongest diffraction maxima. AgCl phase occurrence was reported in our previous article^19^ and Cl presence was later confirmed by EDX analysis. Many chlorine biomass sources are known, including industrial fertilizers and combustion, but there are also natural volcanic and marine life sources^21^. Moreover, studies have also established chlorine content in isolated chloroplasts, and this can have an important role in photosynthesis^22^. Released silver can be transformed to Ag_2_S and/or AgCl inorganic compounds in appropriate salt and/or environmental conditions. Further, Ag speciation in the aqueous environment depends on the Cl-Ag region; where soluble AgCl_2_, (AgCl_3_)^2−^ and (AgCl_4_)^3−^ can be formed from high Cl-Ag ratios, and AgCl can precipitate at lower Cl-Ag ratios^23^. Herein, we consider Ag-AgCl co-existence with a polycrystalline nature. Mixing *Tilia* sp. leachate with aqueous AgNO_3_ led to media colour change from light yellow to dark brown-orange. This colour change confirmed successful synthesis and verified silver presence via the absorption of atomic and molecular electron transitions from ground to excited states. The absorbance maximum was approximately 460 nm (Fig. 1b), and the wide peak indicated different sized Ag NPs in the colloid. TEM analysis confirmed this finding, and also that the presence of organic stabilizers in the *Tilia* sp. leachate influenced the UV-VIS spectrum wide peak.

### Prepared nanoparticles size distribution and morphology

**Figure 2:**
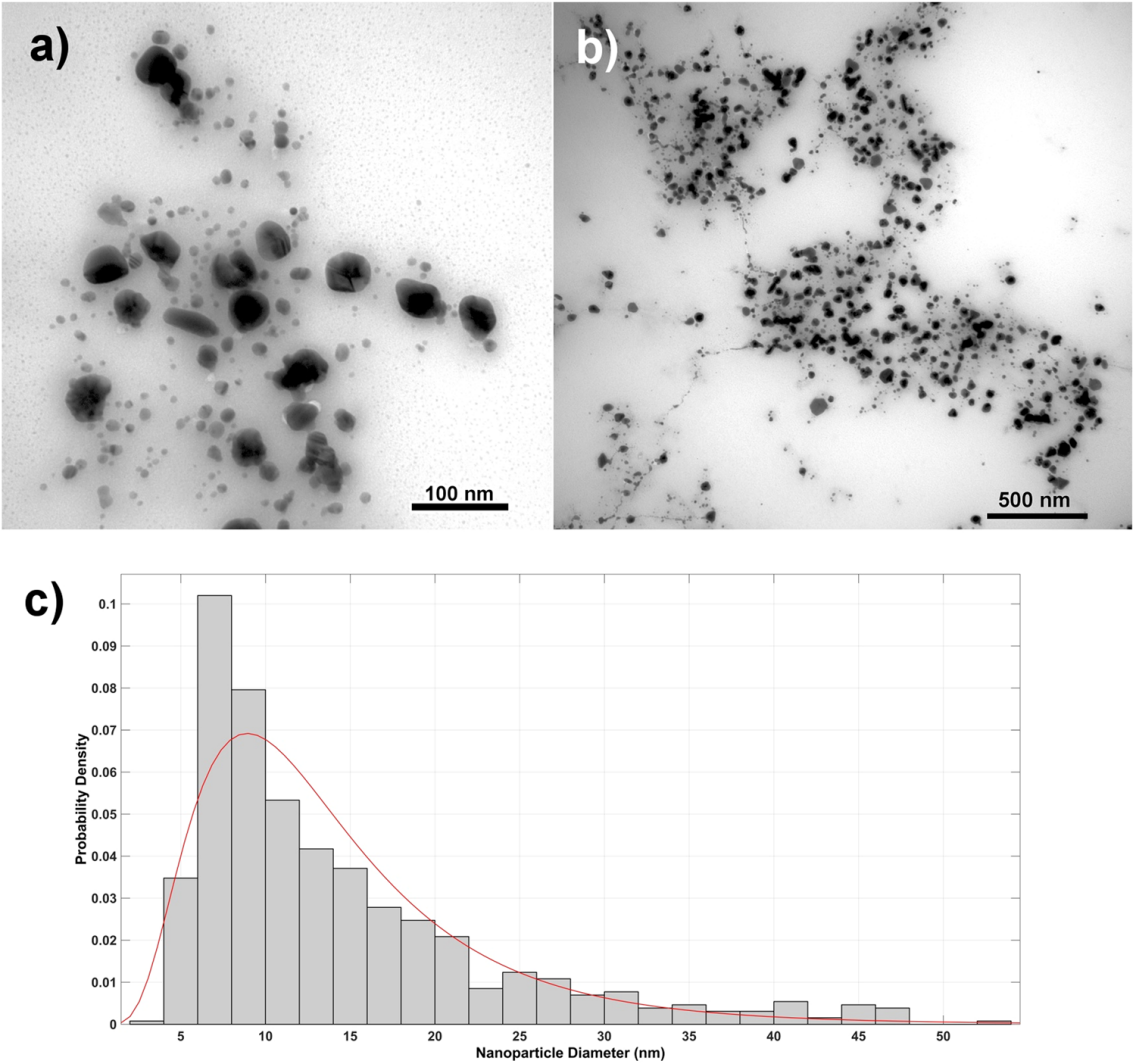
TEM analysis revealed spherical nanoparticles (**a**,**b**) mostly with two size fractions. The 14 nm Ag-AgCl NPs are present in the majority (**c**) however obvious presence of 50–60 nm NPs was also confirmed.

TEM analysis confirmed Ag-AgCl NPs presence and these had mostly spherical shape (Fig. 2). The image analysis determined average NP size at approximately 14 ± 9 nm (Fig. 2a,b). While most values were in the 6–10 nm range (Fig. 2c), 50–55 nm large particles were also detected; and those over 50 nm were excluded from final diameter calculations as these would cause inaccuracy in final mean size. The Ag-AgCl NPs in the colloid formed “clusters” from the presence of organic components in the *Tilia* sp. leachate which formed stabilizing coating (Fig. 2b), but aggregation/agglomeration was not visible. In addition, comparison of DLS and TEM analysis confirmed the influence of organic compounds. The DLS size distribution of Ag-AgCl particles was in the 25–615 nm range, and measurement one week later determined the same size values. While DLS analysis established the average Ag-AgCl NP size at 164 nm, the TEM image analysis provided 14 ± 9 nm “core diameter” average. Based on these results, the DLS analysis determined significant bio-compound influence; and it is widely accepted that this measurement method includes the thickness of bio-compounds on the NPs surface^24^.

### Chemical-physical study of Ag-AgCl nanoparticles

ζ-potential value above ±30 mV usually proclaim a stable system, and stability depends on several parameters. Although the ζ-potential of colloidal Ag-AgCl NPs was determined at −26 mV, the colloidal system did not achieve the theoretical boundary value. However, TEM and visual analysis did not confirm the predicted aggregation and colloidal system stability.

Bhattacharjee (2016) previously recorded that pH provided sufficient/insufficient charge to confirm colloid stability. Herein, the pH value of the pure plant leachate was 5.4 ± 0.1 and this decreased to 4.9 ± 0.1 immediately after mixing the leachate with the silver precursor. The colloid was measured again after 24 hours and pH decreased further to 4.3 ± 0.1; possibly during the NPs reduction process. However, this 24-hour colloid pH value remained constant and this established colloid stability.

### Characterization of input polymer solutions

The conductivity and viscosity of the prepared polymer solutions were examined because altered parameters led to different properties in the final fibrous samples. The PVA-Ag solution had increased conductivity from 322 to 733 μS·cm^−1^ due to the presence of conductive metallic NPs, on the other hand viscosity decreased from 672 to 629 cP (0.672–0.629 Pa·s). The effect on viscosity might be influenced by several factors. Ag-AgCl NP presence in the PVA solution can also lead to NP surface interaction with the hydroxyl group, and Chou *et al*. (2014) reported that the amount of hydrogen bonding between PVA chains can be reduced^25^. Nanofiller can affect the macroscopic properties of the entire material^26^. The greater number of solvent molecules and fewer chain entanglements at low viscosity <0.1 Pa·s increase the surface tension along the electro-spinning jet and cause bead formation along the fibre. When viscosity increases, there is a gradual change in the beads from spherical to spindle-shape until smooth fibres are finally obtained^20^. On the other hand PVA solution viscosity might be also influenced by the organic stabilizers of Ag-AgCl NPs.

### Structural-fibre analysis

**Figure 3:**
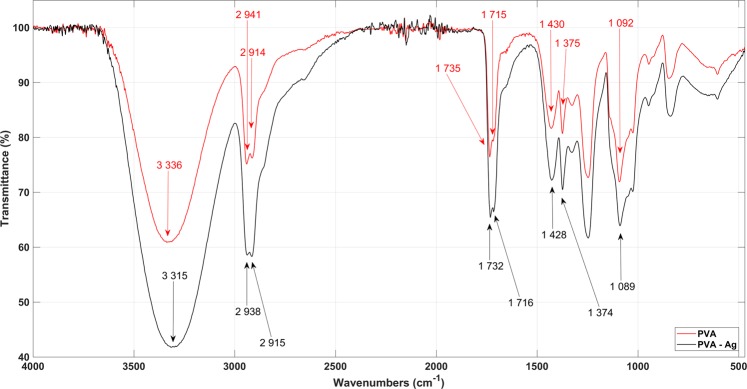
FTIR spectra show functional groups and bonds present in PVA and PVA-Ag samples. No significant differences were observed in PVA and PVA-Ag spectra.

FTIR spectroscopy analysed the PVA molecule functional groups, and Fig. 3 shows the PVA and PVA-Ag spectra. The first intensive peak at 3,336 cm^−1^ is for inter-and intra-molecular O-H bonding and the following peaks were also noted; the peaks at 2,941 and 2,914 cm^−1^ are attributed to the C-H bond from the -CH_2_ alkyl group; those at 1,735 and 1,715 cm^−1^ correspond to C=O bonding; the 1,430 cm^−1^ peak is from the -CH_2_ twisting vibration bond, the 1,375 cm^−1^ denotes C-H and O-H bonds and the last peak at 1,092 cm^−1^ corresponds to C-O-C bonding. No significant difference was noted in the FTIR spectra comparison of PVA and the PVA-Ag mixture, and the single peak shift noted from 3,336 to 3,315 cm^−1^ may have been caused by Ag-AgCl NPs.

### Fibre morphology and diameter distribution

**Figure 4:**
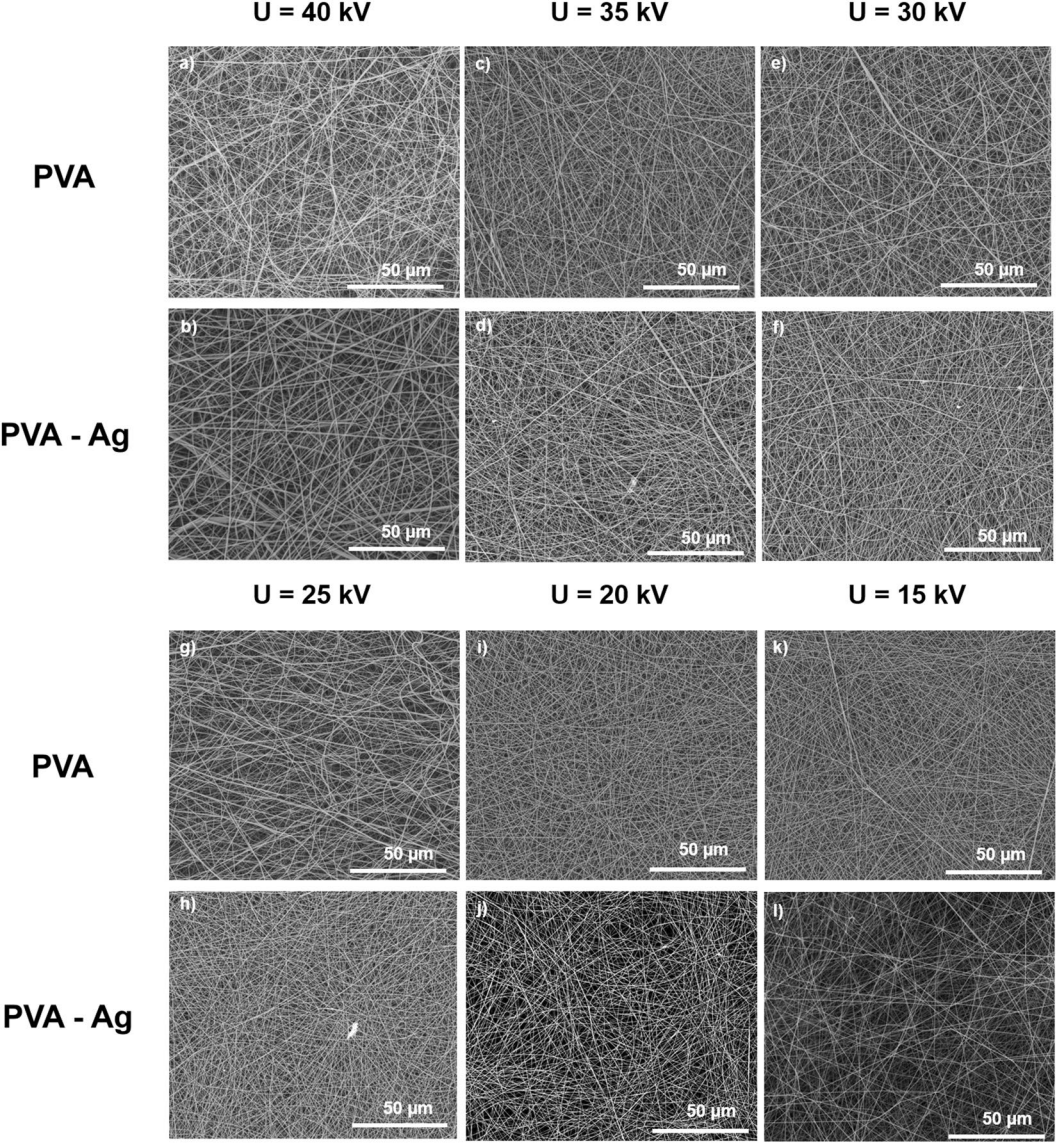
SEM images of PVA and PVA-Ag nanofibrous webs demonstrate the influence of decreasing applied voltage on fiber structure. Micrographs (**a**) to (**l**) show the applied voltage decreases from initial 40 to 15 kV in 5 kV steps.

**Figure 5:**
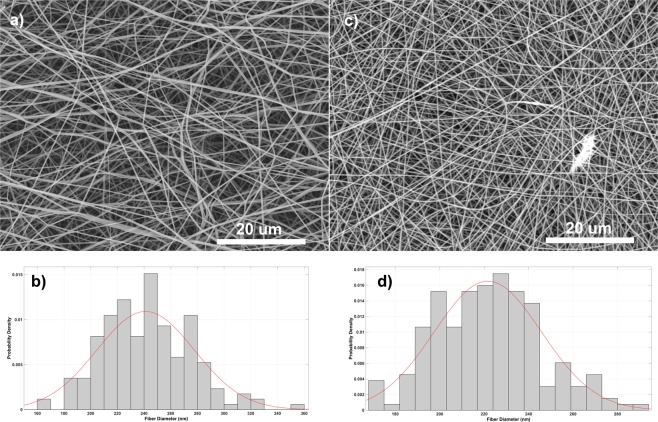
Representative nanofibrous samples and created histograms of nanofibers´ distributions show (**a**,**b**) PVA fibers with average fiber diameters 242 ± 33 nm and (**c**,**d**) PVA-Ag sample with 221 ± 24 nm.

Fibres from the pure PVA solution had the smallest diameters at 40 kV and 15 kV, while the blended PVA-Ag NP fibres were the largest (Fig. 4a-l, Table 1). Herein, we chose two representative samples with 25 kV applied voltage. Figure 5a,c show that the PVA fibre web has 242 ± 33 nm average diameter and lacks a significant number of beads, and also that they were more heterogeneous than the PVA-Ag web with its average 221 ± 24 nm diameter. Moreover, fibre diameter deviation with this applied voltage is less in the blended PVA-Ag than in the pure PVA fibres. While, the PVA fibre SEM images highlighted their smooth surface, higher magnification confirmed that PVA-Ag mixed fibres had NP accumulation which increased the fibre diameter.

**Table 1:** Summary of average fiber diameter and divergence in fibre samples. Samples prepared at 25 kV were chosen as representative samples for further analysis.

PVA			PVA-Ag		
Sample labeling	Applied voltage (kV)	Ø (nm)	Sample labeling	Applied voltage (kV)	Ø (nm)
a	40	217 ± 36	b	40	231 ± 38
c	35	221 ± 31	d	35	228 ± 35
e	30	233 ± 33	f	30	227 ± 37
g	25	242 ± 33	h	25	221 ± 24
i	20	235 ± 27	j	20	224 ± 29
k	15	220 ± 24	l	15	231 ± 34

**Figure 6:**
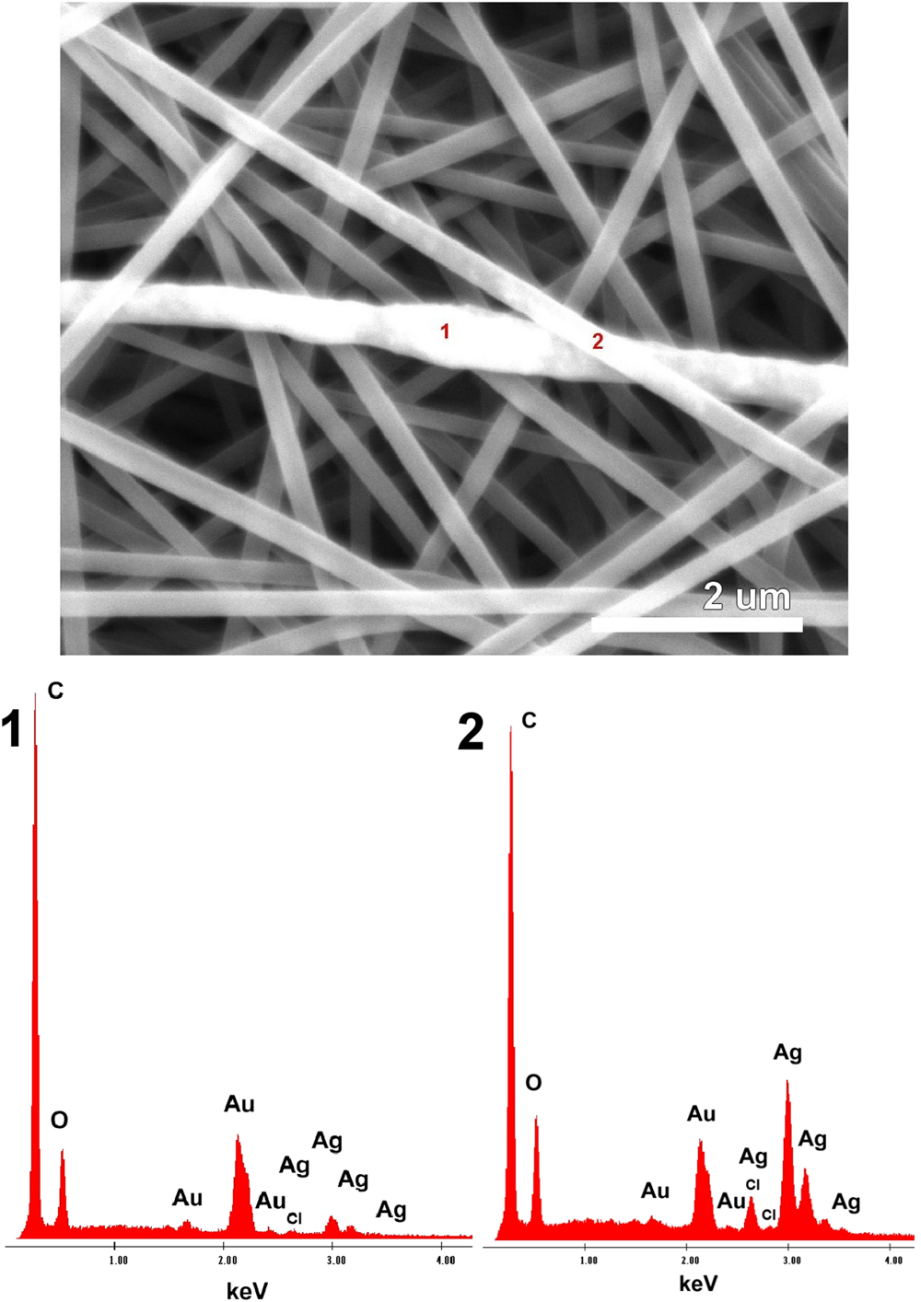
The SEM image indicates Ag-AgCl presence in the PVA structure. EDX analysis shows that the metallic clusters have Ag-AgCl origin; where the chlorine is most likely from initial NPs preparation. Additional detected chemical elements, including C and O, are part of the PVA structure, and the Au presence was due to the necessity to sputter the sample for SEM analysis.

**Figure 7:**
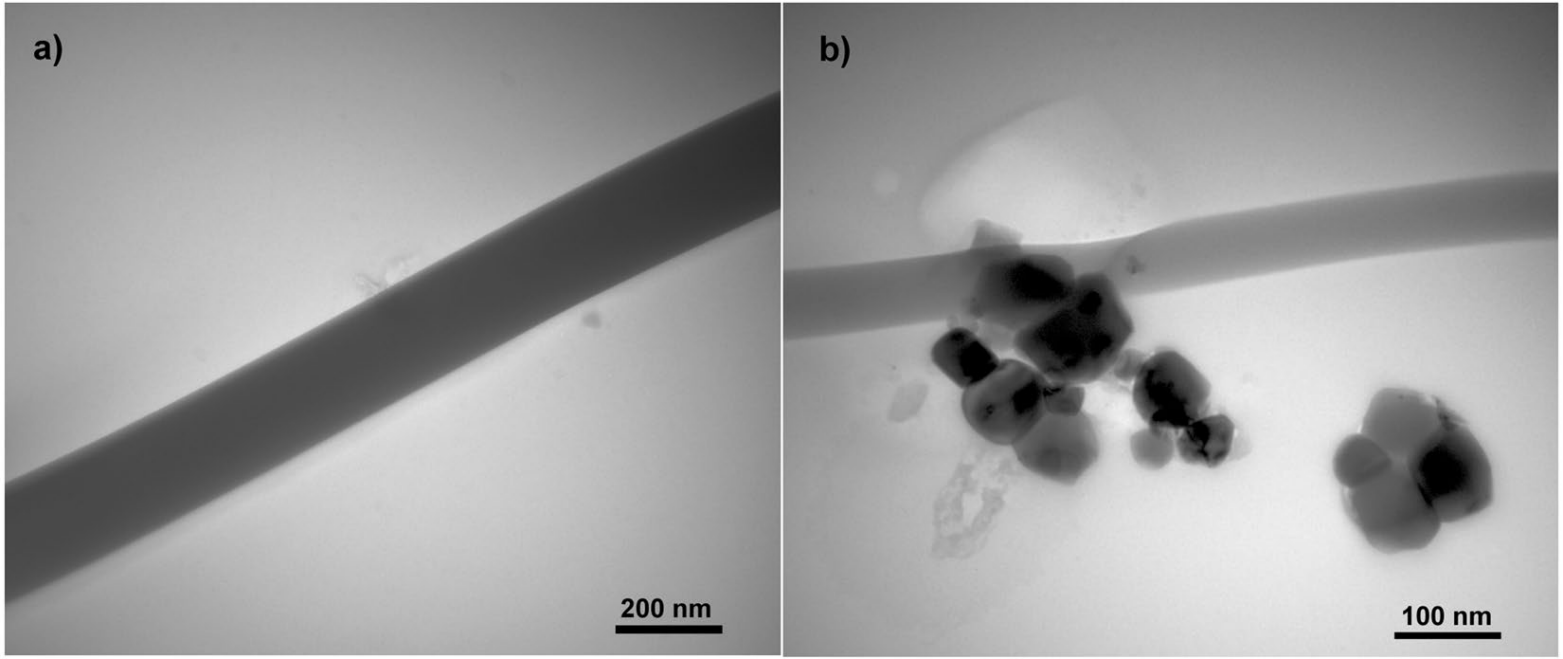
TEM images of PVA and PVA-Ag samples. No NPs are observed in the pure PVA sample (**a**), however the Ag “clusters” are successfully incorporated in the fiber net (**b**). Partial aggregation during polymer solution preparation and the electrospininning process could occur.

SEM and EDX analysis confirmed Ag-AgCl NPs incorporation in PVA nanofibers (see points 1 and 2 on Fig. 6a). The creation of random localized clusters was most likely due to application of the entire electrospinning process, and the location and amount of Ag-AgCl in the sample clusters was easier to identify when lower voltage was applied. Additional detected chemical elements, including C and O, are part of the PVA structure, and the Au presence was due to the necessity to sputter the sample for SEM analysis (Fig. 6b). The presence of Cl correlates with XRD analysis and it likely originates from the *Tilia* sp. biomass. Figure 7 depicts TEM detection of Ag-AgCl NPs in the PVA fibres. The Ag-AgCl NPs were approximately 40 nm, and this corresponds with previous Ag-AgCl NPs results (Fig. 7b). Although partial aggregation could occur during polymer solution preparation or the electrospinning process and larger nanoparticles are spun more easily, we succeeded in final NP integration despite cluster creation. The PVA fibre diameters were approximately 190 nm (Fig. 7a) and fell within the range of SEM analysis. PVA-Ag nanofibers also corresponded to SEM analysis and calculated standard deviations, with diameters of approximately 180 nm (Fig. 7b). The reduced number of nanofibers on the TEM micrographs is most likely due to the preparation of polymers fibres by direct electrospinning for 90 seconds which guaranteed a thin layer of polymer nanofibers available for successful TEM observation. Therefore, future extension of spinning time should lead to the preparation of samples with higher NP frequency and precise detection of their distribution.

### Quantitative analysis of silver in the fibrous material

The experiment was conducted in triplicate to monitor metal exposure in the PVA membrane structure. Results verified that silver is relatively homogeneously spread throughout the fibre structure on the 2 × 2 cm area in the following manner: 0.303 ± 0.018 wt. %, 0.282 ± 0.017 wt. %, and 0.281 ± 0.017 wt. %. Although this reproducibility was confirmed, metal concentration can slightly vary and this must be considered in future applications; including their use in antibacterial coatings.

## Methods

### Phytosynthesis of silver nanoparticles

Ag-AgCl NPs were synthesized using flower-clusters from the *Tilia* sp. deciduous tree collected in the Czech Republic. We have used our optimised previous protocol followed Konvičková *et al*.^19^ and we increased initial AgNO_3_ (Sigma-Aldrich, USA) concentration to 0.01 mol.dm^−3^ and the final colloid was maintained at 4 °C. Briefly, linden “flower-clusters” were dried at laboratory temperature for 5 days, and 1 g of the dried plant and 50 mL of hot (80 °C) DEMI water were then used in linden leachate preparation. Plant was immersed in hot water for 5 minutes and then filtered through membrane filter (0.2 μm pore). Then purified leachate has been mixed with silver precursor in 1:1 ratio.

### Preparation of fibrous samples and the electrospinning process

The following two polymer solutions were prepared prior to the electrospinning process; (1) A 10 wt. % PVA solution (Mowiol 18–88, M_w_ = 130,000 g·mol^−1^, Sigma-Aldrich, USA) was diluted in DEMI water and (2) a 10 wt. % solution of solid PVA from the same producer was diluted in an Ag-AgCl NP colloid to provide the final PVA-Ag solution. These mixtures were stirred for 24 hours at 50 °C to obtain a liquid solution. Fibre samples were prepared by 4SPIN (Contipro, Czech Republic) using Ø = 0.8 mm needle and collected by static continual collector, with 18 cm collector-emittor distance and 20 μl/min fixed flow rate. The 20 minute spinning regime was maintained for each sample, and 4SPIN continuously measured the temperature with 0.4% deviation and 2.5% relative humidity. Table 2 shows changes in applied voltage, temperature and humidity for subsequent nanofiber morphology.

**Table 2:** Summary of changed parameters during the PVA and PVA-Ag sample electrospinning process. The following variables were monitored during this process: changes in applied voltage (kV), temperature (°C) and humidity (%).

PVA				PVA-Ag			
Sample labeling	U (kV)	T (°C)	Φ (%)	Sample labeling	U (kV)	T (°C)	Φ (%)
a	40	22	27	b	40	24	25
c	35	22	26	d	35	24	26
e	30	23	25	f	30	24	24
g	25	23	25	h	25	24	24
i	20	23	25	j	20	24	24
k	15	23	25	l	15	24	25

### Structure and phase analysis

Colloidal Ag-AgCl NPs was characterised by two-beam UV/VIS spectrophotometer Cintra 303 (GBC Scientific Equipment Ltd., Australia). XRD analysis was performed by Bruker D8 DISCOVER diffractometer (Bruker AXS) equipped with an X-ray tube with rotating Cu anode (λ = 1.5418 Å) operating at 12 kW. All measurements were in parallel beam geometry with a parabolic Goebel mirror in the primary beam. The samples were prepared in thin powder layers fixed to a glass plate, and X-ray diffraction patterns were recorded in grazing incidence in the 20 to 80° 2θ angular range, with 0.05° step-size and α = 1.5° angle of incidence. The advantage of this method is that the measured intensities and precise position of the diffraction maxima are not sensitive to surface irregularities and roughness. The Ag-AgCl NPs colloid was centrifuged (Centrifuge 5702, Eppendorf, Germany) and slowly dried at 36 °C for 24 hours. This procedure was repeatedly performed to obtain a representative sample for XRD measurement. Fibrous samples were characterized by FTIR (Nicolet 6700 FT-IR spectrometer, Thermo Nicolet, USA). The operating unit was fully automatic in Omnic computer software. Spectra were measured in the 400–4,000 cm^−1^ range and modified by ATR correction, automatically modified baseline and removal of CO_2_ bands. The samples were subjected to ICP-AES analysis to detect the amount of silver in the fibrous structures by Ciros Vision ICP-AES spectrometer (SPECTRO Analytical Instruments Inc., Kleve, Germany). Approx. 2 × 2 cm PVA-Ag samples were adjusted to a determination of the total metal content.

### Chemical-physical methods of sample characterisation

The ζ-potential of Ag-AgCl sample was controlled by ZetaSizer Nano–ZS (ZEN 3600; Malvern Instruments Ltd., UK) and calculated from the intensity of light scattered by moving particles. This movement was driven by an applied electric field on a cell designed for ζ-potential measurement which established system electrostatic stability. Accompanying pH changes in the NP solution were measured by EcoScan Series pH 5+ immediately after mixing and after 24 hours. The viscosity of homogeneous polymers was then measured by VISCO BASIC Plus viscometer, and dynamic viscosity determined from the magnitude of the torque measured in the steady-state rotation set at 50, 60 and 100 rpm of the appropriate shaft (R4) in the sample. This enabled calculation of average viscosity. Finally, polymer solution conductivity was measured by COND 730 WTW series INO LAB at 23 °C laboratory temperature, and the standard potassium chloride of KCl, C = 0.1 mol·dm^−3^ enabled final value re-count based on the measured standard value.

### Morphology and size distribution

The morphology and size distribution of Ag-AgCl NPs and polymer fibres were characterised by transmission electron microscope JEOL 1011 (Jeol, Japan); with 2 μl of sample placed on copper grids and dried. The biosynthesised nanoparticle size distribution was then evaluated by the JMicroVision program. A minimum of 650 NPs in TEM images were analysed and the resultant histogram was created by MATLAB software. The size distribution of Ag-AgCl NPs in colloid was also evaluated by dynamic light scattering (DLS) by ZetaSizer Nano–ZS (ZEN 3600; Malvern Instruments Ltd., UK), and DLS analysis was conducted in a plastic cuvette cell with 2 mL NPs solution and 1% measurement deviation. Both pure PVA and PVA-Ag fibres were characterised by JEOL 1011 microscope (Jeol, Japan), and grids with nanofibers were prepared by direct electrospinning for 90 seconds. Obtained data was compared with scanning electron microscope (SEM) analysis through FEI Quanta FEG 450 using Secondary Electron Detector (SED), Back Scattered Electron Detector (BSED) and energy dispersive analysis spectroscopy (EDX). Finally, electrospun fibre size distribution was evaluated by the JMicroVision programme. A minimum of 150 sample fibre diameters were monitored in SEM image analysis and resultant histograms were created by MATLAB software. Arithmetic mean and standard deviation has been processed using standard mathematical formulas in MATLAB software.

## Conclusions and Outlook

PVA-Ag investigation continues in the biomedical field because of its successful application in wound dressing. PVA is normally used as a matrix for incorporating inorganic NPs or antibiotics, because it is a water-soluble synthetic polymer^27^ and this inspired our incorporation of NPs in a fibrous matrix using their initial natural environment. This required preparation by phytosynthesis in *Tilia* sp. aqueous solution, and it was then followed by determination of the silver concentration in the prepared solid fibrous samples.

We succeeded in this method of crystalline Ag-AgCl NPs preparation which retains their properties during processing and combination with other materials. This especially involves maintaining morphology, size (14 ± 9 nm) and conductivity, although TEM determines only a small part of the total sample. We achieved successful incorporation in the uniform electrospun 221 ± 24 nm poly(vinylalcohol) fibres and both SEM with EDX and TEM analysis determined fibre uniformity with the presence of silver nanoparticles. ICP-AES confirmed the relatively similar metal concentration throughout the triplicate measurement of fibre structures on the 2 × 2 cm area in the following manner: 0.303 ± 0.018 wt. %, 0.282 ± 0.017 wt. %, and 0.281 ± 0.017 wt. %. An important advantage of this method is the ease of nanoparticle release and activation for further application through PVA solubility.

In conclusion, our study provides a solid foundation for the following future research: (1) determination of the influence of inorganic elements such as Cl on Ag-AgCl nanoparticle morphology and structure; (2) optimization of the electrospinning process to avoid creation of nanoparticle clusters and improvement in the final products’ properties; (3) the choice of appropriate antibacterial testing of water-soluble materials and (4) final confirmation of fibrous materials’ antibacterial activity, and extended Ag-AgCl nanoparticle use in medicine.
